# Exploring informed consent in HIV clinical trials: A case study in Uganda

**DOI:** 10.1016/j.heliyon.2016.e00196

**Published:** 2016-11-14

**Authors:** Agnes Ssali, Fiona Poland, Janet Seeley

**Affiliations:** a Medical Research Council/UVRI Uganda Research Unit on AIDS, P.O Box 49, Entebbe, Uganda; b University of East Anglia, Norwich NR4 7TJ, UK; c London School of Hygiene and Tropical Medicine, Keppel Street, London WC1E 7HT, UK

**Keywords:** Social sciences, Health sciences, Medicine

## Abstract

**Introduction:**

In settings with low literacy levels ensuring that participants are fully-informed before they consent to participate in clinical trials is a challenge. We explored the experiences and concerns of key actors in the informed consent process in two HIV clinical trials.

**Method:**

Semi-structured interviews were conducted with 46 respondents including trial participants, research study team and research ethics committee members about their experiences during the informed consent process. Three focus group discussions were conducted with 14 Community Advisory Board (CAB) members and 17 trial participants. Data were analysed to identify key themes.

**Findings:**

The consent process was highlighted as an important procedure by all the key actors however each group had a particular area of emphasis. Signing a consent form was given importance by research team and ethics committee members, because it provided documented evidence of a participant’s willingness to join a clinical trial. Participants did not welcome the presence of a witness for a non-literate participant because understanding study information was not closely related to an ability to read and write.

**Conclusion:**

This study’s findings indicated that obtaining a volunteer’s signature or thumbprint on a consent form did not necessarily mean that the participant was fully-informed about the information relevant to their taking part nor that they understood all the information shared with them. Informed consent requires sufficient time in the research process to have staff trained well enough before research begins. Ensuring and gaining informed consent should be understood and treated as a relation-centred, dynamic supportive process throughout the duration of a research study.

## 1 Introduction

Requiring participants to sign a consent form is currently expected practice in clinical trials as a way to ensure participants can be seen to have freely agreed to take part in a trial as the best judges of their own interests [1]. However what the different actors experienced during the informed consent procedure is usually not discussed. Researchers have reported that voluntariness may be attained by participants within a complex context which will include political, cultural, social and economic factors [2] which may not reflect researchers’ and regulators’ assumptions. This paper focuses on the perceptions of the different actors in the informed consent process on their experience of managing the procedure of signing a consent form in two HIV clinical trials in the Uganda context. The actors in the informed consent process examined here include: the research team, the trial participants, the Regulatory Ethics committee (REC) and the Community Advisory Board members (CAB).

Informed consent is usually seen as incorporating four components: disclosure of information, the prospective participant’s comprehension to make an informed decision, the participant’s being free from coercion, and a participant’s giving consent explicitly and formally usually in written form ([3], [4], [5], [6]). In this paper, the term ‘informed consent' will be used to refer to researchers giving full information about what is involved in conducting an HIV clinical trial and ensuring that the person receiving the information has the capacity to make a decision and is voluntarily consenting to take part when he/she is satisfied that they have understood the information [3], [7], [8]. Therefore, in this paper, as well as receiving information on the study and signing the consent form, the ‘informed consent process’ includes, all the interactions that occur during a research process which inform a participant about the study.

Formal records of informed consent are widely seen as formal evidence that a volunteer’s autonomy was respected and that they made an autonomous decision to take part in the trial [9], [10], [11]. The primary goal of ensuring informed consent is to protect participants’ welfare and respect their individual autonomy [12]. This involves recognising a person’s capacities and perspective on what happens to them during research, including accepting their right to hold certain views, make certain choices and act based on their personal values and beliefs [7], [13].

‘Autonomy’ is a challenging concept which can be interpreted differently by different disciplines. Appelbaum et al. ([14] (1987: 22) define it as ‘personal freedom of action or the right to do as one pleases within certain restrictions’. However, personal freedom to make decisions is sometimes affected by the environment in the community where the individual lives; because decision making in most African communities is embedded in social relations and influenced by culture, gender and values upheld in a particular setting [15, 16, 17]. The public health facilities and health services in Uganda are still insufficient for the general population; there are few doctors and nurses to take care of the huge population and drugs are not always available in the public health centres [18]. However, the research clinics which conduct research are part of the environment but the clinics are usually better-equipped with the necessary facilities and have well-trained health workers who are usually economically more advantaged and have better education levels than most of the research participants. In a study conducted in South Africa, the researchers found that sometimes patients relinquish their autonomy and depend on the health workers decisions about their health because they think health workers are competent in what they do [19].

International and National Human Research Guidelines require that consent be formal and documented [1], [20], [21] as evidenced by a participant/patient’s signature on a consent form. Findings from a demographic health survey in Uganda showed that there are still some Ugandans that have either no formal education or only primary level education. The proportion of female and male with no education increases with age; for example, 12% of women aged 25–29 have never attended school, compared with 59% of women aged 60–64 [22]. Given the high percentage of the population who cannot read and write, the process of getting signatures is unlikely to be easy. Although literacy is not equivalent to actually comprehending study information, it influences the consenting process because it affects the involvement of both the participant and a witness.

In this paper, we discuss the views of different actors in the informed consent process and their experiences of informed consent. Specific reference is made to the procedure of signing a consent form and the role of a witness during this process. We explore views and experiences of respondents with participants who are non-literate. Here we use the word “non-literate” only to mean someone being unable to read or write, not as being otherwise limited in their wider understanding, since limits in literacy skills cannot be assumed to limit understanding. The findings inform the day to day practice of informed consent in HIV clinical trials.

## 2 Methods

### 2.1 Study design

The present study was a qualitative exploratory research project nested within two HIV clinical trials, and it sought to explore what happens when implementing the informed consent process within these clinical trials. The respondents were purposively selected to include the key actors in the informed consent process in the two HIV clinical trials.

### 2.2 The trials in which this study was nested

Trial One was a phase I double-blind placebo-controlled trial to evaluate safety and immunogenicity of two HIV vaccines with HIV-1 uninfected adult participants. The participants in the trial were adults aged 18–40 years.

The second trial, investigated a Phase IV randomised trial evaluating the safety of discontinuing treatment with Cotrimoxazole (septrin) prophylaxis among HIV-infected adults on ART in Uganda. The participants were adults aged 18–59 living with HIV who were already stabilised on ART and had a CD4 of over 250 cells.

### 2.3 Study area

The research was situated at two Medical Research Council/Uganda Virus Research Institute (MRC/UVRI) Uganda Research Unit on AIDS sites. One of the trials was in a semi-rural area and second trial was conducted in a semi-urban area. Both sites were in the central region of Uganda and are about 139km apart.

At the Trial One site, most of the population were involved in peasant farming producing cash crops including maize and coffee. Trial Two was situated near the shores of Lake Victoria where the population were mainly fisher folk involved in, fishing and selling fish, food business and running small kiosks were the other main activities. Both areas have been affected by HIV, with some participants currently living with HIV and others having lost close family members, friends and neighbours to AIDS.

### 2.4 Selection of respondents for the qualitative study

There were five categories of respondents in this study who were purposively selected in relation to their having different roles in the informed consent process comprising: the research team (counsellors, nurses, clinicians, mobilisers and senior researchers), the Research and Ethics Committee (REC) members, the Community Advisory Board (CAB) members, the trial participants and their spouses or close friends. This purposive selection therefore enabled an understanding of the informed consent process from the different perspectives relating to these key roles. In this paper we do not present findings from spouses or close friends because there were very few interviews in this category and those interviewed focused on the spouse or friend’s support to trial participants.

Research Ethics Committee (REC) members were included because they approve the trial protocols and the participant information sheets and consent forms. The REC is responsible for monitoring approved studies. The REC members were approached by the first author, who discussed the study and requested for appointments for the interviews. The first author conducted the interviews with each of the selected members after they consented to take part in the study. The REC was represented by 5 members from different disciplines which included: statistics, public health, social science, clinical/epidemiology and a community representative.

The research team members were selected for this study because they implement the research protocols and conduct the trial procedures. They have the greatest interaction with the participants who join the clinical trials. The research team included the senior scientists who were co-investigators and coordinators of the trials and the study team for each of the trials included counsellors/health educators, nurses, clinicians and mobilisers. The research team was approached after the investigators had agreed to the inclusion of the trials in the qualitative study. Each of the research team members was approached individually to seek their consent for the interview.

The Community Advisory Board (CAB) members were included in this study because they advise on research taking place in their community. They are a link between the community and the research team. The CAB gives feedback to the research team concerning what is happening in the community and they also inform the community members they represent about what is happening at the research centre. CAB members were approached through the community liaison officer and they individually consented to take part in the study by signing a consent form. The CAB members were invited to participate in a focus group discussion. The CAB had representatives from the district medical office, health centres around the research unit, other HIV and AIDS research and service providers in the community, religious leaders (Christian and Muslim) and local community leaders.

The trial participants included in this study were those who were already taking part in the two HIV clinical trials and consented to take part in the qualitative study. The inclusion criteria for participants in the qualitative research required that:

• the trial participants had to have been in the trial for at least 6 months,
• they were willing to give additional time in addition to the time spent in trial activities
• they were willing to be interviewed at least three times for this study every three months
• they were willing to sign a consent form to participate in the qualitative study.

Participants were first approached by the research nurses in the two clinical trials since they were already involved with the participants. The nurses informed the participants briefly about the qualitative study which was to be nested within the clinical trial. The participants, who were ready to hear more about the qualitative study, met the first author and discussed the study information and then each volunteer who consented to take part in the interviews and focus group discussions signed a consent form. When a volunteer consented to take part, he or she was interviewed and the next appointment for the second interview was made and at the second interview, a third appointment was made. In both trials follow up was every three months, so this project fitted in with that schedule. Two participants, a male and a female, declined participation because they did not want to give additional time for research.

### 2.5 Data collection and analysis

To gain an understanding of how the different actors in the two HIV clinical trials, experienced and managed the informed consent process, the first author (AS) conducted semi-structured in-depth interviews and focus group discussions with them. The interviews and focus groups were audio-recorded. The first author moderated the FGDs with a research assistant. The first author is a Ugandan and the FGDs and interviews were conducted in the vernacular language common in the two research sites. The respondents in this study were all aged 18 years and above. The research team and the REC members were interviewed once by the first author and the interviews were conducted in English apart from the community representative where the local language and English were used.

The trial participants were interviewed three times by the first author, the first interview was conducted when they consented, and the second and third at three monthly intervals. Data collection lasted for one year from January to December 2012. See Table 1 for categories of respondents and data collection methods.

Data were downloaded from the digital recorder each day and then transcribed and saved on a secure drive. The transcripts were first reviewed manually by the first author in order to extract codes. Initial codes were generated from the first six in-depth interviews which included two from the trial participants, two from the research team, and two from the REC members and discussed with the second and third authors of this paper. The initial codes were then applied to the whole data set [23], [24], [25]. Data analysis was on-going and inductive throughout the data collection process. Data analysis was aided in later stages by the use of NVivo 8 qualitative software in order to manage the coding.

### 2.6 Ethics and permissions

This study was reviewed and approved by the Science and Ethics committee (SEC) of the Uganda Virus Research Institute and the Ethics committee of the School of International Development at the University of East Anglia (UEA) in Norwich in the United Kingdom. It was cleared by Uganda National Council for Science and Technology (UNCST). The respondents were involved in clinical trials and permission was sought through the different trial steering committees and the site principal investigators before the respondents were approached.

All the interviewees gave individual consent to be interviewed by signing a consent form. To preserve anonymity, while reporting findings, we refer to Trial One or Trial Two as the trial to which each respondent contributed, and use pseudonyms so as not to reveal actual names of respondents.

The results presented in the following sections cover responses from the different categories of actors in the informed consent process: the trial participants, the research team, the REC and the CAB. The results are presented for all the actors in relation to the sub sections: demographic characteristics, roles in the consent process, comprehension of study information, significance of signing a consent form, views on using thumb prints and witnesses and suggestions to improve the informed consent process.

## 3 Results

### 3.1 The demographic characteristics of the study respondents

A total of 63 respondents took part in this study. The selection of the research team, the research and ethics committee members and the community advisory board members depended on their roles in the trial. The demographic characteristics of the research team, ethics committee and the community advisory board members were not collected in this study. See Table 1 for respondents in this study.

The research team was composed of professionals who included scientists, nurses, counsellors/health educators, clinicians and a community mobiliser. The REC members were professional and a community representative who lives in one of the communities where one of the trials was being conducted. The CAB had members who were professionals and lay community and religious leaders. The trial participants had various skills ranging from professional to vocational as presented in Table 2 which shows their demographic characteristics. See Table 2

The mean age of the trial participants in this qualitative study was 32.6 (33) years, ages ranging between 19–50 years. All the participants reported that they had attained some level of education but for the majority this was limited to the primary school level. As was reported in the Uganda national literacy statistics where female are less literate than male [22], we found a similar pattern with the participants in this study, 11 (91%) out of the 12 who had primary education (first seven years of schooling) were female; and some of these had some difficulty writing their names and their reading was quite slow even in the vernacular. The female participants were mainly doing casual work which in this context pays less money than formal employment. The male participants in this qualitative research had low income paying jobs and the university under graduate was not yet employed.

The participants in Trial One were in regular sexual relationships reflecting the inclusion criterion for the trial and each of the participants had a spouse who had been tested for HIV. In the qualitative study, this was not a criterion and the participants were recruited as individuals. The participants in Trial Two had varying marital status: married, separated, widowed and single because the main inclusion criteria for the second trial did not involve a sexual partner. The inclusion criteria for the participants to join the trial were that they had tested HIV positive and also met the other inclusion requirements of that trial.

### 3.2 Roles of the different actors in the informed consent process

As part of understanding informed consent, respondents were asked to discuss the roles of the actors in the informed consent process. All respondents described the trial participant as central to the informed consent process:

*I think the person at the centre is the volunteer [participant], because … [one,] this is somebody who is supposed to come in willingly; two, he has rights which he may not be aware of and yet these rights should not be compromised at any time and without him or her no study can take place, even if you have all the money.* (Robert, scientist, Trial One)

The participants often displayed a precise awareness of their contribution to the process:

*My role is important because if we don’t enrol then who will they conduct the research on? Will they do it on animals? So that all shows that the research team has to handle us well.* (Susan, participant, Trial Two).

Another participant explained the importance of her involvement as a way of encouraging other people to join research:

*My main responsibility is to avoid getting HIV and to discuss information that I have with other people … after this experience when I meet people and there is research being done, I encourage them to join.* (Fiona, participant, Trial One)

The CAB members reported that their role was to support the research team in the selection of trial participants. They said they are the mobilisers in the community.

REC’s involvement in the informed consent process was more involving at the time of protocol review before the trials were implemented. After the approval of a given protocol, they are less directly involved in managing the consenting procedure. The REC is however mandated to carry out monitoring visits to the sites where approved trials are conducted.

### 3.3 Views on the comprehension of study information by trial participants

Researchers have tried out several ways to try to assess volunteer understanding which include quantitative tests and narratives, but it remains challenging to ensure participants understand study information [9]. When discussing comprehension of study information between the literate and non-literate participants; most participants reported that it did not matter whether they signed or thumb-printed on a consent form; what was important was the potential participant being able to understand the information given by the research team. A number of trial participants highlighted that participants ‘comprehension’ and ‘signing consent form’ were two different aspects of the informed consent process as one of them put it:

*We need to know that both a person who has understood and one who has not understood can sign.* (Doreen, participant, Trial One)

This quote indicates that at least some participants saw it as possible for participants, whether they could write or were only able to thumbprint the consent forms, to join a trial without understanding the preliminary trial information.

The research team members indicated that although they may have found it easier to deal with a participant who could read and write and so sign the consent form easily without requiring a witness, they did not see those who could not write as necessarily understanding the study information less well:

*There are participants that never got the opportunity to go to school, but they have common sense and they will reason things out much better even than those who have been to school, so I do not see the difference …* (Aida, clinician, Trial Two)

For the REC members what was critical was that a participant understood the study information and was protected by the researcher from social and physical harm as has been outlined in guidelines [26]. The REC members mentioned the importance of voluntary participation. They noted that participants’ understanding of the study information should not be linked to their ability to read and write, because even those who could not read could understand information if it was provided to them in simple clear terms.

CAB members noted that although signing of the consent forms was very important to the researchers and was seen to imply that the individual had understood the study information; in this community if the influential people in the community such as religious leaders and some cultural leaders endorsed an activity the majority of people would follow what their leaders have approved. Such communal decision-making would then be based on the beliefs and values of these influential persons rather than individually-informed decisions, similar to what has been reported elsewhere [27]. Religious leaders were singled out by the CAB members as very influential and trusted by the majority of the people in the communities.

### 3.4 Perspectives on significance of signing and thumb printing the consent form

Conducting clinical trials requires following informed consent guidelines most of which require that trial participants sign a consent form before getting involved in any trial [21, 28]. Each of the actors was asked what the significance of signing or thumb printing a consent form meant.

All the trial participants in this study noted that it was important that they sign or thumbprint the consent forms and that documenting this informed consent was also needed to indicate that they had joined the research freely. While discussing the reasons why they needed to sign a consent form, the majority of the participants argued they did this to show that they had not been coerced to join the research and it would protect the researcher in case a volunteer experienced side-effects.

Most trial participants discussed signing or thumb printing the consent forms matter-of-factly, as a usual procedure, perhaps indicating that they were used to the way research studies are run by the MRC. That however, might mean that they actually considered signing a consent form before they consider the key trial messages discussed with the trial research teams at the start of a trial. One male participant asked the first author (AS) to let him sign a consent form even before they discussed the study information sheet because he explained that he trusted the researcher.

The research team suggested four main reasons why it would be important for participants to sign the forms: participation without coercion, legal agreement, accountability and the protection of researchers. However each of the research team members emphasised one or two of these aspects in relation to their role in the informed consent process; the nurses, counsellors and mobilizer saw signing the consent forms as significant in providing evidence to formally demonstrate that the participants were participating in a study without being coerced and had understood the information the research team had given them.

Senior researchers described a signed consent form as protecting them from being suspected of having coerced a participant:

*I think they need to sign, or else someone can easily deny that they agreed to participate in your data. If they say they did not agree, what evidence do you have to show that they actually agreed to participate in your study? (*Festo, Scientist, Trial Two)

The clinicians and scientists described the signed consent forms as evidence of the discussion of the research information that happened between the potential participant and the researcher. The signed consent form would also confirm the existence of an agreement between the researcher and the participant about taking part in a research.

One of the clinicians noted that a researcher who signed the consent forms was taking responsibility within the research study for keeping the participant from physical and social harm and ensuring the participant’s confidentiality.

One research team member said that signing the consent forms served both the researcher and the trial participant; “*For the researcher, the signed consent form was a record to show that the volunteer participated freely and for the participant they were a record that they could show to others to demonstrate their participation in research*”. However, sharing of records was not common for all the participants. During the focus group discussions, some participants reported that they had kept their involvement in research as a secret because of the stigma attached to research which was about HIV and AIDS.

The signed forms provided evidence for the researcher to the regulatory authorities and study monitors that the participant had been informed about the risks of the trial. However, one of the scientists remarked that the signed consent forms did not reveal to someone outside the process what had transpired between the researcher and the participant during the individual information sharing session:

*It is a sign that something has happened. It does not tell you* what *happened; it does not tell you the details of what happened … it does not tell you whether I gave the information very well; it does not tell whether the person understood everything; it just tells you yes, there might have been a process and you know the two people signed.* (Robert, scientist, Trial One)

These findings confirm that signing or thumb printing the consent forms as discussed by the research team was an obligation set for them by the regulatory authorities which the research team had to fulfil if they were to be seen to adhere to the national and international ethics research guidelines [1], [21].

The interaction and trust built between the two people who signed the consent forms, the research participant and the researcher was reflected in the related discussion as more important in encouraging on-going participation of the participants in a trial than the documents they signed, even though the latter provide documented evidence.

The focus group discussion with CAB members suggested that one of the main reasons for participants signing the consent forms was to protect the research team from being sued in courts of law for not communicating the right information to participants about possible negative effects of the trial products. They saw a signed consent form as a source of legal cover for the researcher:

*… if a [participant] has a problem along the way they cannot take you to court because they agreed … For example if someone in the vaccine trial got another disease they couldn’t just blame the organization.* (CAB, FGD)

The CAB members commented that signing the consent form implied that the signatory had received the study information and had agreed to be part of the study, whether the trial result was positive or negative. CAB members discussed that signing a consent form was useful for the research team when following up participants because some may have similar names and the signatures help show the difference.

CAB members compared signing or thumb printing a form to a land-buying transaction, which was common in this community. The CAB members speculated that if nothing was signed to show that there was an agreement between the researcher and the participant, this may have meant that no discussion of the study information had taken place between the research team and the participant. A CAB member quoted an expression commonly used in the community to express this:

*We have a certain project working on quality improvement, and we have a slogan that says ‘anything not documented is not done’. However much you may work, until you document it is not done. If you gave verbal consent you didn’t write it [and so it is not done*] (CAB, FGD).

### 3.5 Views on thumb printing on a consent form

In this particular research context, usually a number of research participants are unable to read or write on documents, so they have to thumb print on the consent form in the presence of a witness. A recent Uganda National Household survey showed that the overall literacy rate of Ugandans aged 10 years and above, was 71%, with men being more literate at 77% and women at 65% [22]. This meant that adopting differing processes for achieving and recording informed consent according to literacy levels might lead to varying experiences of informed consent.

While discussing views on whether to sign or thumb print a consent form, the participants suggested it might give them a higher status among their peers if they could sign the consent forms with their names, rather than putting a thumb print on it. A male participant mentioned that thumb printing may be used in banks, but did not see it as acceptable to ask a research participant to put their thumb print on the consent forms, when they were able to write their name, because this meant they were literate and therefore had the ability to sign on the consent form. Some of the participants, however, said it would be acceptable to them to either sign or thumb-print on a consent form as long as they had understood the trial information.

Four of research team members interviewed saw it as generally easier to deal with literate than with non-literate participants during the information sessions and consenting procedure. The research team said this was because where a thumbprint had to be used they needed to find someone to witness the participant’s thumb printing, making the process more complicated for the research team to manage signing:

*[Signing] shows an independence − that I have read this myself and I am signing it … For those who thumbprint there is that traditional element: you must have a witness because you read to them and then someone has to thumbprint and the witness signs … So it introduces another person, and you usually have to make sure they understand what this person understands, and this adds another dimension.* (Robert, Scientist, Trial One)

However, this quote also illustrates how introducing a witness for a non-literate participant during the consent process could reduce the participant’s autonomy as a third person was introduced into their discussion with the research team member. The participant lost some of their independence to make their own decision because the witness had to be present while they were being given information and when they thumb printed to join the research. It also added to the research team member’s workload because they also had to ensure that the witness had understood the study information before they signed for the participant.

REC members argued that thumb printing was not a disadvantage to the trial participant. One committee member commented that it would be fine if all participants thumb printed on the consent forms because no one could forge or lie about another person’s thumb print.

### 3.6 Views on the role of a witness in the informed consent process

The presence of a witness is particularly required by the Ugandan regulatory authorities when a non-literate potential participant is being given information about the study by the research team and during the consent procedure [20, 21]. The presence of a witness during the consenting procedure is intended to ensure that the non-literate participant’s autonomy was being respected by the researcher. The witness had to ensure that the participant they were witnessing for understood the study information and agreed to the requirements of a trial before they gave their consent.

Participants attached stigma to thumb printing, mainly because it involved the presence of a third person as a witness. During the focus group discussion, the participants mentioned that an adult who was not able to write might well find it embarrassing to get a witness among those from the same community, some of whom may be looking up to him as an elder. Social stigma being enacted in these community settings has also been reported elsewhere and it can affect potential participants [29].

The research team saw the issue of providing witnesses to the informed consent process as difficult to manage, both for the participant and for the researcher involved in facilitating their consent. Challenges were identified by some research team members from their experiences of the consent process for participants. They observed that participants might not want anyone other than the researcher to know that they could not sign or write their name. They identified challenges for them in managing the research process from having to involve two people, the non-literate participant and his or her witness to obtain consent ensuring that both understood the study information. This requires communicating successfully with both individuals.

Ideas about who should act as a witness to a non-literate participant’s consent varied among the research team. Whereas some felt it was all right to use any witness as long as the participant agreed, even if they did not previously know the witness, some thought that the witness should be someone the volunteer knew:

*The best would be a person close to the participant, someone who cares, a person who loves the person, has known this person for some time, or a person who benefits from the welfare of this patient to the extent that if the patient is disadvantaged due to participating in research, then this person is also disadvantaged because of that.* (Timothy, Health educator, Trial Two)

The appropriate witness for a non-literate participant was therefore often described as a person who would scrutinize the study information on their behalf and could analyse the benefit and risks to the participant. The health educator noted that a witness involved during the consenting process might not be answerable to the participant if the latter was harmed during the research; in such a situation the witness’s responsibility would be seen as ending with the signing of the consent forms [12].

Some research team members felt that the participants were disempowered by their reliance on the study team to pick a witness, although they may have agreed to or refused to have the person proposed. The partial loss of the research participant’s decision-making power could also affect their self-determination later in the research project [14].

The research team found the process of securing witnesses for non-literate participants to be onerous because they had to include a witness for each non-literate participant before they could proceed with the consent process. This was even more challenging if the witness was a fellow-participant who would be free to choose to opt out or withdraw from the trial at any time. In such a situation, the research team had the view that it would impact on the non-literate participant’s continued participation in a trial, since the witness was seen to be a support for the non-literate participant. In contrast, the research team members said that if a witness was a neutral person who was not a close relative and was not in a trial, they might be much less likely to understand what a participant went through in taking part in a trial and might not give appropriate social support. These were important issues for the research team to achieve the participant’s autonomy in the informed consent process.

REC members explained that because they did not supervise the informed consent process directly it was difficult for them to confirm that there was a witness who also understood the study information before signing for a non-literate participant. The committee had to trust the researchers’ reports about consenting participants.

### 3.7 Concerns reported during the informed consent process

The research team noted that as well as the challenges in involving a witness for the participants who could not read or write the research teams reported having to manage a different type of problem with another group of participants, who did not really know how to read and write properly but insisted on signing the consent forms. Their written signatures appeared different on the screening consent form and the enrolment consent form because they were just learning to write their names.

Some other elements of informed consent which were of concern to the research team included: getting an adequate thumb print, suspicion of the consenting procedure for research by some participants who did not understand why they had to sign or thumbprint if they had already verbally agreed to take part in a trial, explaining technical terms in lay terms, and strict time lines which gave the research team little time to fully understand study information before they shared it with the participants.

The challenge of getting thumb printing right was noted by a clinician:

*You roll your thumb from one side to the other, but [participants] become kind of stiff, they just make a small spot, and this one is kind of twisted, … In the end when you compare their thumbprints they don’t look the same.* (James, clinician, Trial Two)

Although participants agreed to participate in a trial some research team members reported that participants usually joined expecting one specific procedure and that it could be difficult for the researcher to get a participant to understand the other procedures before consenting, to take part in the trial:

*In my experience, during the informed consent process when you give people information about what you are studying they lock onto something and … that is what they use to join your study. You are going to tell them ‘we are going to do A, B, and C’ − they lock onto something, maybe A, and it is what they base joining your study on.* (Festo, scientist, Trial Two)

Some research team members found it difficult to explain some of the factual content on the study information sheets to the research participants for example:

*We had about four meetings … so that we could understand … There is a paragraph which states: ‘… it is not known whether your risk of acquiring HIV infection will increase, decrease or remain the same’; that statement really confused us, and the participants.* (Sylvia, nurse, Trial One)

The CAB’s main concerns about the informed consent process were to ensure the participant from the community was protected from both social and physical harm. Their emphasis on the presence of “important persons”, like the religious and cultural leaders in the community, who may influence decisions is an important aspect for community engagement.

Table 3 sets out a comparison of responses from the different actors in the informed consent process.

## 4 Discussion

This study investigated perceptions and experiences of the different actors in the informed consent process with particular reference to participants’ comprehension of study information, significance of signing a consent form, issues about providing a thumb print and the involvement of a witness in the consenting procedure. The national and international consent guidelines require that research participants give written rather than oral consent [1], [20, 21]. This is intended to ensure that the potential participant has understood the study information relating to the aim and objectives and decides freely to take part in the research. We found that all actors including the research participants in this qualitative study viewed the signed consent form as a legal document binding the researcher and the trial volunteer in an amicable relationship for the duration of the trial.

Trial participants did not mention information related to study content, such as the trial objectives and procedures, as reasons for signing a consent form, despite this being one of the basic reasons for ensuring informed consent. While a signed consent form met the stipulations of informed consent guidelines, the form recorded nothing about what was involved when researcher and participant discussed the procedures of a trial. The form therefore gave no indication of the volunteer’s comprehension of study information. While testing comprehension is given explicit importance in clinical trials, the “when” it is done will also be critical as noted by one participant. If testing for comprehension of study information was too soon it may have reflected that a participant is able to recall words but not with certainty that they comprehended what information the researchers gave them.

The study revealed that there could be varying reasons why participants signed a consent form. If participants were only concerned with signing the consent forms as a prerequisite of joining a trial, this could mean that some of them did not understand the implications of being a research participant in a randomised clinical trial. While the researchers viewed the form as a literal basis for evidencing participant autonomy in the informed consent process the participants saw it more as a document that they signed like a contractual agreement to join the research study.

There are limits of the consenting process that a signed consent form can represent as noted by one scientist since its outcome was so dependent on the interaction between the researcher and the volunteer who sign the form. The discussion on how to engage a participant until they made their decision to join a trial may not be clear to the person who was not part of the interaction. He/she will not know what was discussed, what was agreed upon and the kind of questions that were asked by the participant before they agreed to sign the consent form.

This raises an important ethical issue for the “informed” aspects of “informed consent” because the trial information given to the potential participant may only have been limited in explaining scientific terms as reported by some participants. This may imply that some participants may join a clinical trial without having understood some aspects of the trial.

An ethical dilemma identified in this study relates to that arising from the varying literacy levels of the participants in this context. In this setting, having non-literate participants was not uncommon and therefore assessing participant understanding of study information entailed several ethical dilemmas. These included assessing volunteer understanding, individual and community stigma attached to thumb printing on a consent form and the presence of a witness during the consenting procedure for non-literate participants.

While using uniform tools to assess comprehension may have been important as suggested by many researchers, the main contributing factor to participants’ understanding study information and key concepts is researchers using a language that the participants would understand in the relevant context. This is similar to the reports by Brehault et al., [30] that when information sheets were translated into the local language of the participants, communication between the researcher and the participant was made easier. Afolabi et al., [31] conducted a study to review how informed consent comprehension is defined and measured in African research settings and found that study participants’ comprehension of key study concepts was poor, suggesting a need to develop a uniform definition for informed consent comprehension after developing appropriate tools. This is indicated in our findings that the trial participants requested that information discussed with them needed to be simple and clear to enhance comprehension.

Our findings show that in this context there was stigma attached to thumb printing a consent form rather than signing it off. In communities like these, where not everyone in the community has had an opportunity to go to school, some individuals might be exposed to stigma attached to those not able to write one’s name. Researchers might be less aware of this risk. A genetic study conducted in Ethiopia recommended the need for investigators to seek to assess and address risks of research as perceived by the prospective participants [29]. The participants while not experiencing challenges with comprehending study information, commented emphatically on the problems their fellow participants experienced with thumb printing [28]. Failure to write one’s name on a consent form currently means the presence of a witness will be routinely required during the consenting procedure according to the regulatory ethics guidelines [1], [21], [26], [28], [32]. While this may have solved a researcher’s and ethics committee formal problem; it would be likely to raise many other challenges for researchers and participants’ work and status.

Although signing or thumb printing was always carried out at the start of a trial, the majority of respondents, including the trial participants, asserted that this also affected the volunteer’s self-determination and autonomy, often for the duration of the study [33]. Thumb printing by all participants in research could reduce the stigma faced by the non-literate participants in trials.

The research team and ethics committee members also assumed that the witnesses ‘understood the information better’ than the non-literate participants and could also help them understand it. They nonetheless noted that obtaining a thumbprint from a participant took more time than a signature and that the presence of the witness made the participant uncomfortable when they were thumb printing the consent form.

The differences noted in perceptions of the different research team members around what qualified a witness for a non-literate participant suggest this may require more ethical discussion about the obligations that should be attached to the role and who should be judged an appropriate witness in a given research context.

Participant confidentiality and privacy during the research was less certain in circumstances that involved a witness. Including a third party in the agreement between the research team and the non-literate participant violated the latter’s privacy according to some research team members. Even when they had understood the study information the non-literate participant was also being required to relinquish part of their power to make their own decision to take part in a trial, to the witness at the signing of the consent form.

Of all the issues covered by the informed consent guidelines, the presence of a witness when a non-literate participant formally consented to join the trial aroused the most controversy in the individual interviews of all the actors and during the focus group discussions, and yet this was a requirement laid down in the national and international guidelines [1], [20], [21], [28].

Our findings show that participants may join a trial after deciding to focus mainly on just one aspect of the trial and to rely on their relationship with the research teams. Other study findings in East Africa found that a participant may sign a consent form because of the trust they have for a research member or focusing on one aspect of the procedures of a trial [34], [35], [36], [37], [38], [39], [40], findings which are similar to our findings in this study

Additional resources may be needed to build the capacity of research teams to manage this requirement to consent participants, especially those who are non-literate if they are to address the extra safeguarding and trust building activities by researchers required as reported in this study. A similar concern was expressed during a workshop on informed consent [41].

## 5 Limitations

The main study limitation was that there could have been some selection bias in terms of who chose to take part in the qualitative study. However, the participants were in a similar stage of their clinic scheduled follow-up visits and all the other respondents took part in this study because of their role in the clinical trial. Although the qualitative study was conducted with trial participants who had been in research for at least six months, no observation was therefore made at the time of their entry to the trials and the researcher could have missed the first time experience of participants at the inception of the trial. The first author obtained individual informed consent from all the respondents taking part in this qualitative study.

## 6 Conclusion

It could be seen in this study that obtaining a participant’s signature or thumbprint on a consent form did not necessarily mean that the participant who signed a consent form was either fully-informed about the information relevant to their taking part nor that they also understood all the information communicated to them by the research team. The presence of a witness during the consenting of a non-literate participant could also be seen to have put participants in a more- rather than a less-vulnerable social position within the research process. When involving non-literate people, the formal ethical requirements in the informed consent process could be seen as subjecting such participants to stigmatising processes right from the start of a research trial, while a participant was thumb printing while giving consent, to the end of their involvement in the trial. Such potential for stigma may mean higher risk that participation is not wholly voluntary. Thus, while the formalised process of obtaining a signature and thumb print from a potential participant may work successfully, in terms of gaining written record of consent the study findings also show that the procedure may cause humiliation for the non-literate participants which may adversely affect participants who are non-literate. Ensuring and gaining informed consent should therefore be understood and treated as a dynamic, relation-centred, supportive process throughout the duration of a research study to facilitate participant autonomy.

## Declarations

### Author contribution statement

Agnes Ssali, Fiona Poland, Janet Seeley: Conceived and designed the experiments; Performed the experiments; Analyzed and interpreted the data; Wrote the paper.

### Funding statement

This work is jointly funded by the UK Medical Research Council (MRC) and the UK Department for International Development (DFID) under the MRC/DFID Concordat agreement. We thank the MRC/UVRI Uganda Research Unit on AIDS who funded the first author’s PhD.

### Competing interest statement

The authors declare no conflict of interest.

### Additional information

No additional information is available for this paper.

## Figures and Tables

**Table 1:** showing data collection and category of interviewees.

Activity	Study Site	Category of respondent	No. of respondents	Gender
In-depth interviews (audio-recorded)	1	Senior scientists Counselors Nurses Clinician Community mobiliser Trial participants	2 2 3 1 1 13	M M, F F M M 7M, 6F
In-depth interviews (audio- recorded)	2	Senior scientist Counselors/health educator Nurses Clinicians Trial participants	1 2 2 2 11	M M, F F F, M 3M, 8F
In-depth interview(audio recorded)		REC	5	3M, 2F
Focus group discussions (audio recorded)	1 2	Trial participants CAB Trial participants	9 14 7	4M, 5F 7M,8F 1M, 6F
Short interview		Spouses	4	3M, 1F

**Table 2:** Demographic characteristics of the Trial volunteers in this study.

Demographic characteristic	Numbers	
Sex	9 male, 14 female	
Age	Aged from 19–50 years	Mean age is 33 years
Marital status	Married 10(4F,6M) Single 7(4F,3M) Widowed 3 Separated 3	
Education level	Primary level 12(11F,1M) Secondary 4(2F,2M) Vocational 5(1F,4M) University 1M	
Occupation	Small business- 12(11F,1M) Students 2(1M,1F) Formal employment 2M Casual worker, 5(3M,2F) House work (2F) Unemployed (1F)	Saloon, roadside selling, shops University, vocational Security officers Driver, School cleaner, motor vehicle mechanics

**Table 3:** Comparing responses from the different actors.

Actor	Role in trial/informed consent process	Overall values	Definition of informed consent	Views on consent form	Views on thumb printing/witness	Recommendations
Research team	Give study information to volunteer obtain consent from volunteer −conduct study procedures	Value is to recruit and follow up according to protocol	Main emphasis is about information giving, agreement and signing of consent forms	Helping volunteer to understand design of trials not easy signing of consent forms may be viewed suspiciously −volunteers join research for usually one specific procedure a volunteer whether literate or not may/may not understand study information Volunteers do not necessarily understand information at the start of a trial	−some find dealing with non-literate volunteers difficult in communicating information-the practice of getting the thumbprint is difficult-some believe a witness can be anyone others say it should be a close relative/friend	− involve literate family members in the consenting process-train research teams on the importance of informed consent-REC should do spot checks-community engagement should start early in communities where volunteers are to be selected from-the literate peers among the volunteers could participate in information sharing sessions-volunteers need at least a day to two weeks to study the study information provided to them before they are requested to sign on the consent form-need for on-going discussions between the actors(SEC,CAB, Volunteers and research team)
Volunteer	−take part in the research-inform other community members and interest them in research	−value is attached to altruism for some and for some it is about what benefits particularly related to their health well being	−main emphasis is that there are no risks to the individual and benefit of health care that may be lacking in the public health care clinics/hospitals	− it shows a volunteer has accepted to take part in research-by signing a consent form it does not mean that one has understood the study-if any one refuses to sign then it is an indicator of refusal to take part	−thumb printing is stigmatised in community	−understanding of study information should be gauged after they have been in a trial for some time to avoid recall bias volunteers need more than a single information session-information given to volunteers should be clear and easy to understand-interventions to support literacy and numeracy should be developed
REC	Ensure the set national and international research guidelines are operating at the research sites-review protocols and informed consent documents-monitor what takes place at the research sites	Value is attached to the effort of ensuring volunteer do not experience any form of harm (social and Physical)	Emphasis is on follow guidelines and safeguard volunteer from any form of harm (physical, social, emotional)-use simple and clear language in consent forms	−it is the only way to confirm that a volunteer agreed to participate in a trial-signing or thumb printing is not a problem as long as forms give full information and no harm done to volunteer-do not relate volunteer’s understanding to ability to read and write	The guidelines require this to safeguard the volunteer-signing and thumb printing is currently the only way the process is documented and should continue	−consent forms should be short-contact information should reflect researchers at the sites-emphasise verbal information before you introduce documentation-introduce visual aids like flow charts to describe informed consent process-protect interests of the community
CAB/community	The main link to the study population in the community-gatekeepers to the community, they support initial contact with potential volunteers	Value is attached to respect for the individual and the community	Emphasis is on free choice for volunteer to take part or refuse to take part-no coercion	− signing consent forms is to protect the research-signing implies volunteer has accepted to take part in the research	−if information is provided in a language a volunteer understands, whether one signs or thumb prints both are capable of understanding information, so it does not matter	−The activities of CAB should be monitored to encourage accountability-need frequent meetings with research team-they want a more active involvement as stakeholders in the community
